# ﻿The Araceae of Sumatra I: A new species of *Alocasia* from Aceh, Indonesia

**DOI:** 10.3897/phytokeys.249.133737

**Published:** 2024-11-21

**Authors:** Ni Putu Sri Asih, Ina Erlinawati, Yuzammi, Julisasi Tri Hadiah

**Affiliations:** 1 Research Center for Biosystematics and Evolution, National Research and Innovation Agency (BRIN), Jl. Raya Jakarta - Bogor KM. 46 Cibinong, Bogor, West Java, Indonesia Research Center for Biosystematics and Evolution, National Research and Innovation Agency Cibinong Indonesia

**Keywords:** Aceh Besar, *Alocasiaroseus*, Indonesian flora, new taxon

## Abstract

*Alocasiaroseus* is described as a new species from Aceh Besar Regency, Sumatra (Indonesia). The plants found produce stunning inflorescence, leading to over-collecting in the wild. The new species is morphologically similar to *A.flemingiana* but differs by its greyish green adaxial leaves and pale reddish to greenish purple or pale brownish green abaxial leaves, lacking interprimary veins and sinus not naked, thecae overtopped by synconnective, and a pale pink appendix. The new species is also similar to *A.arifolia*, from which it differs by the glabrous and pale dull green petiole, the colour of abaxial and adaxial leaves, not forming interprimary vein, sinus not naked, and pale pink appendix. The new species is compared with other similar Indonesian taxa and an identification key to the species of *Alocasia* in Sumatra, supplemented with photographs, are provided.

## ﻿Introduction

The genus *Alocasia* (Schott) G.Don (Araceae Juss.) consists of 100 species (Promprom et al. 2024) but current research suggest that there may be an additional 41 undescribed species (Boyce and Croat 2023). This genus is distributed in tropical and subtropical Asia ranging from the Malesian region toward Oceania and to mainland Australia (Nauheimer et al. 2012; POWO 2024; Promprom et al. 2024). Borneo is considered to bear the richest diversity and endemism of *Alocasia* (Hay 1998). However, the diversity and distribution of *Alocasia* are poorly understood in the Indonesian archipelago, with about 27 known species (Asih and Lestari 2022; Asih et al. 2022). Prior to this study, there were seven species of *Alocasia* recognised in Sumatra (Hay 1998; Erlinawati 2011; Kurniawan et al. 2013; POWO 2024).

Our knowledge of *Alocasia* in the Sumatra is currently inadequate and, consequently, further exploration and collection of plants is necessary. The under-collection of *Alocasia* from Sumatra is reflected in the few specimens held at Herbarium Bogoriense (BO) and other international herbaria. Furthermore, the last taxonomic revision of *Alocasia* was done over twenty-five years ago (Hay 1998) who recognised six Sumatran *Alocasia* taxa (*Alocasiaalba* Schott, *A.arifolia* Hallier f., *A.inornata* Hallier f., *A.longiloba* Miq., *A.kerinciensis* A.Hay, and *A.puber* (Hassk.) Schott). *Alocasiamacrorrhizos* (L.) G.Don is listed as an introduced species to Sumatra (POWO 2024).

Based on Praetorius s.n. (L.1415481), *A.puber* was thought to occur in Sumatra (Hay 1998), but is here now regarded as *A.alba*. *A.puber* is only known from Jawa. Erlinawati (2011) recorded *A.flemingiana* Yuzammi & A.Hay from Siberut Island (a small island off the western coast of Sumatra), a species that was previously only known from Jawa (Hay 1998). A recent study by Mustaqim and Setiawan (2019) provided an update on the distribution of *A.alba* upon finding the species in Tanggamus Regency, Lampung Province, Sumatra.

Since *Alocasia* has been known as one of the most popular ornamental plants, sought for and traded by enthusiasts and the general public, many species are collected directly from the forest and traded illegally. One of the species, traded by people from Aceh, is here considered as the new species *Alocasiaroseus*. The work is part of an ongoing study of Araceae family in Sumatra region.

## ﻿Material and methods

**Figure 1.**
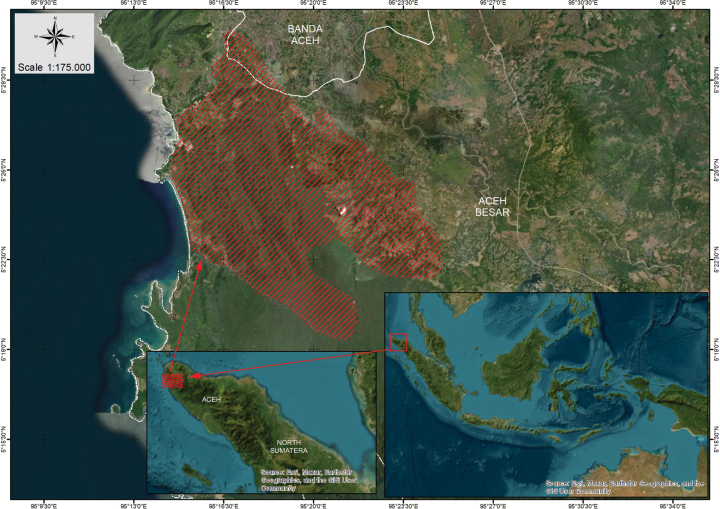
Distribution map of *Alocasiaroseus* Asih & Yuzammi, sp. nov. showing the location where the new species was discovered in Aceh Besar (red shading). Inserts: overview of Indonesian archipelago (right) and Aceh Province (left). Map by Rani Yudarwati.

Plants were collected from the forest near Kueh Kemukiman Keude Bieng, Kueh Village, Aceh Besar Regency, Aceh Province, Sumatra (Fig. 1), by people who live nearby the forest. It was then cultivated at a nursery, from which we obtained some plants. Pertinent literature was considered for the taxonomic investigation (e.g. Hallier 1901; Hay 1998) Photographs of herbarium specimens held at L were included in this study.

## ﻿Taxonomic treatment

### ﻿ Alocasia roseus

Taxon classificationPlantaeAlismatalesAraceae

Asih & Yuzammi sp. nov.

207F41EE-6FAC-57C7-B508-A4BA33BB9CD5

urn:lsid:ipni.org:names:77346080-1

Fig. 2 , Table 1

#### Type

**Figure 2.**
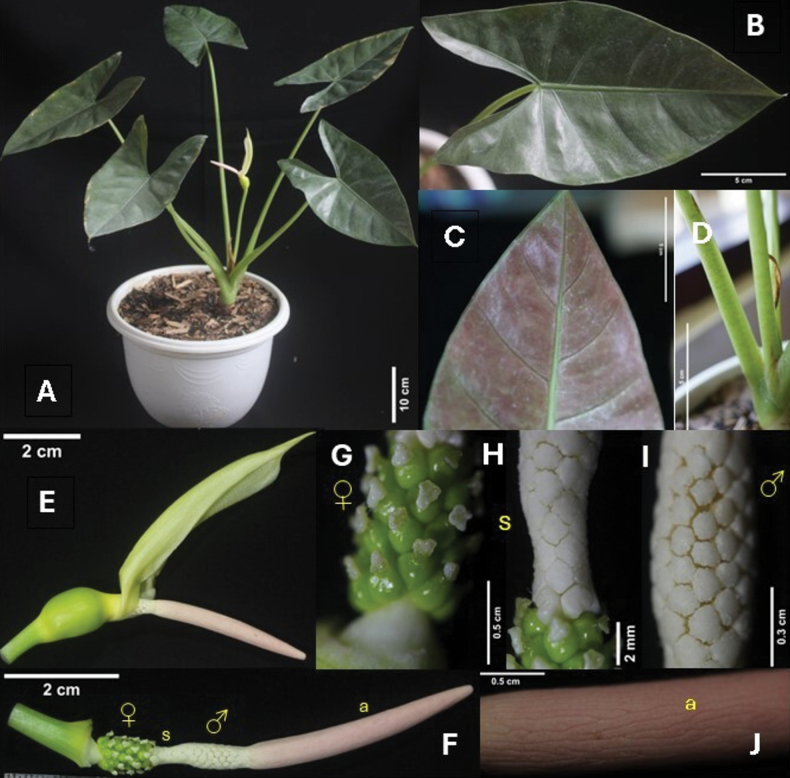
*Alocasiaroseus*: **A** habit **B** adaxial leaf **C** abaxial leaf **D** petiole **E** inflorescence with spathe **F** inflorescence with the spathe removed showing the zonation of reproductive organs **G** female zone (♀) **H** sterile interstice zone (s) **I** male zone (♂) **J** appendix (a). (Photographed and edited by Ni Putu Sri Asih and Julisasi Tri Hadiah).

**(prepared from a cultivated plant in a private nursery).** Indonesia • Aceh, Aceh Besar, Lhoknga, Desa Kueh, Kueh Kemukiman Keude Bieng, *PSA 395* (holotype BO! [dried specimens and inﬂorescences in spirit]; isotype ANDA!) (Fig. 2).

#### Diagnosis

**(Table 1).***Alocasiaroseus* is morphologically similar to *A.flemingiana* and *A.arifolia* from which it differs by having greyish green adaxial leaves and pale reddish to greenish purple or pale brownish green abaxial leaves, interprimary vein absent, sinus not naked, and pale pink appendix (*vs.* mid-green to dark green adaxial and green-yellowish/paler abaxial leaves, forming poorly to conspicuous interprimary vein, sinus naked up to 3 cm, and cream/pale apricot appendix).

**Table 1.** Comparison of important diagnostic characters of *Alocasiaroseus*, *A.flemingiana* and *A.arifolia*.

Diagnostic characters	*Alocasiaroseus*	*Alocasiaflemingiana*	*Alocasiaarifolia*
Blade	leathery, greyish green adaxially, reddish to greenish purple to pale brown greenish abaxially	membranous, mid-green adaxially, yellowish green abaxially	membranous to thinly coriaceous, somewhat glossy, dark/mid-green adaxially, paler abaxially
Venation	not forming interprimary collective veins	interprimary veins absent or poorly differentiated	forming undulating to zig-zag interprimary collective veins
Glands	inconspicuous axillary glands	distinct small flat glands	inconspicuous axillary glands
Sinus	not naked	naked for up to 1 cm	naked in the sinus for up to 3 cm
Constriction	at base of male zone or to half way along male zone	at top of sterile zone of spadix	at sterile interstice
Spadix	shorter than spathe, shortly stipitate for 2–4 mm long	somewhat shorter to almost as long as spathe, stipitate for *c.* 5 mm	almost as long as spathe, shortly stipitate, *c.* 3 mm long
Thecae	overtopped by synconnective, thecae opening by apical pores	somewhat displaced to overtopped by synconnective, thecae opening through apical slits	overtopped by synconnective, thecae opening by apical pores
Appendix	pale pink, gradually tapering to a blunt point, faintly irregularly channeled	Cream-coloured appendix, tapering	pale apricot-coloured, somewhat constricted at base, slightly narrower than male zone, tapering toward a point

#### Description.

Small herb c. 55 cm tall; ***rhizome*** erect; ***leaves*** several together; petiole 34–38 cm long, pale dull green, glabrous, faintly mottled greenish, sheathing in the lower about 1/3 of its length, pale dull green; ***blades*** leathery, sagittate, greyish green adaxially, reddish to greenish purple to pale brown greenish abaxially, margin entire; ***anterior lobe*** 12.8–14.4 cm long, 9.7–11.6 cm wide, the widest is base of anterior lobe, tip acuminate, 7 mm long; anterior costa with 4–5 primary lateral veins on each side, proximal ones diverging at 76–96° on each side then running to submarginal vein, distal primary veins diverging at 40–45° on each side, primary vein prominent adaxially then forward to marginal becoming flush to lamina, prominent abaxially, with inconspicuous axillary glands, secondary and tertiary venation flush to lamina adaxially, rather prominent and conspicuous abaxially then running to conspicuous submarginal vein inserted c. 1.5 mm from margin, interprimary collective veins absent; ***posterior costae*** diverging at 60–80°, not naked in the sinus; ***posterior lobes*** acute, 6–7.2 cm long; ***inflorescences*** soliter or in pair, subtended by green cataphylls and then dried at flower anthesis; ***peduncle*** to c. 14.6 cm long, resembling petioles in colour and faintly mottled; ***spathe*** c. 9.7 cm long; ***lower spathe*** ovoid, yellowish green, c. 2.3 cm long, c. 1.8 cm diam; ***limb*** lanceolate, yellow to greenish, erect then tilted 45° after 3 days, separated from the lower spathe by a constriction at the base of male flowers or at top sterile interstice (to the midpoint of the male zone); ***spadix*** shorter than the spathe, c. 7 cm long, shortly stipitate, 2–4 mm, whitish-green, cylindric; ***female zone*** cylindric, 0.9–1 cm long, 0.9 cm wide; ***ovaries*** subglobose, green, stigma raised on a style 0.5–1 mm, conspicuously, (2–)3–4-lobed, pale yellow; ***sterile interstice*** cream-coloured, not attenuate, 0.4–0.5 cm long, narrower than male zone, with 4–5 whorls of rhomboid synandrodia; ***male zone*** cylindric, cream-coloured, 1.1–1.2 cm long; synandria rhombohexagonal to somewhat irregular, with the synconnective overtopped the thecae; thecae opening by apical pores; ***appendix*** pale pink, gradually tapering to a blunt point, faintly irregularly channeled, ***c***. 3.5–4.2 cm long, slightly wider than the male zone; ***fruit*** unknown.

#### Distribution and habitat.

The species is only known from Aceh Besar, Sumatra Island. It grows on the hillside of the forest, in shade and humid areas.

#### Etymology.

The specific epithet, *roseus*, is based on the pale pink appendix of the staminodes found in this new species. This color of the appendix is rarely found in the genus.

#### Conservation status.

*Alocasiaroseus* is known only from a single locality in the forest near Kueh Kemukiman Keude Bieng, Kueh Village, Aceh Besar Regency, Aceh Province, Indonesia. Since further populations could occur, we prefer to assess these species as Data Deficient (DD) according to the IUCN Red List criteria (2022).

#### Notes.

*Alocasiaroseus* belongs to the informal group “Macrorrhizos” (*sensu*Hay 1998) in view of the following showed characteristics: inflorescences in pairs, the spathe constriction aligning with the sterile interstice of the spadix, and the synconnective overtopping the thecae. This species is the second, besides *A.balgooyi*, in the Macrorrhizos group with no naked sinus in their posterior lobe. *Alocasiaroseus* also has an appendix color that differs from other species in the Macrorrhizos group. Pink being a rare colour of appendix that is found only in *A.melo* and *A.princeps* (Hay 1998).

### ﻿Key of *Alocasia* Species in Sumatra

**Table:** 

1	Leaf blades shallowly to completely peltate in mature plant	**2**
–	Leaf blades not peltate in mature plant	**3**
2	Peltate leaf with posterior lobes almost fully fused; appendix white	***Alocasiakerinciensis* A. Hay**
–	Peltate leaf with posterior lobes never almost fully fused; appendix very pale orange to bright yellow	***Alocasialongiloba* Miq.**
3	Robust to massive plant	**4**
–	Medium to small plant	**6**
4	Interprimary collective vein well-defined	***Alocasiaalba* Schott**
–	Interprimary collective vein poorly defined	**5**
5	Inflorescences paired among lead base; peduncle exceeding length of cataphylls at anthesis; petiole glabrous	***Alocasiamacrorrhizos* (L.) G.Don**
–	Inflorescences many, clustered together; peduncle short, mostly hidden with leaf sheath and cataphylls; petiole glabrous or minutely pubescent	***Alocasiainornata* Haillier f.**
6	Intermarginal vein not formed; petiole puberulent or glabrous	***Alocasiaarifolia* Hallier f.**
–	Intermarginal vein conspicuous; petiole glabrous	**7**
7	Abaxial leaf reddish to greenish purple to pale brown, with greenish tinge; appendix pale pink	***Alocasiaroseus* Asih & Yuzammi**
–	Abaxial leaf yellowish green; appendix cream-colored	***Alocasiaflemingiana* Yuzammi & A.Hay**

## Supplementary Material

XML Treatment for Alocasia roseus

## ﻿Additional information

### Author ORCIDs

Ni Putu Sri Asih https://orcid.org/0000-0002-4161-9235

Ina Erlinawati https://orcid.org/0000-0002-4621-4456

Yuzammi https://orcid.org/0000-0002-9637-9048

Julisasi Tri Hadiah https://orcid.org/0000-0002-5478-8118

### Data availability

All of the data that support the findings of this study are available in the main text.
